# Antiplasmodial activity, in vivo pharmacokinetics and anti-malarial efficacy evaluation of hydroxypyridinone hybrids in a mouse model

**DOI:** 10.1186/s12936-015-1032-5

**Published:** 2015-12-16

**Authors:** Ntokozo S. Dambuza, Peter Smith, Alicia Evans, Jennifer Norman, Dale Taylor, Andrew Andayi, Timothy Egan, Kelly Chibale, Lubbe Wiesner

**Affiliations:** Division of Clinical Pharmacology, Department of Medicine, University of Cape Town, Observatory, Cape Town, 7925 South Africa; Department of Chemistry, University of Cape Town, Rondebosch, Cape Town, 7700 South Africa

**Keywords:** Malaria, Pharmacokinetics, Hydroxypyridone-chloroquine, In vivo efficacy

## Abstract

**Background:**

During the erythrocytic stage in humans, malaria parasites digest haemoglobin of the host cell, and the toxic haem moiety crystallizes into haemozoin. Chloroquine acts by forming toxic complexes with haem molecules and interfering with their crystallization. In chloroquine-resistant strains, the drug is excluded from the site of action, which causes the parasites to accumulate less chloroquine in their acid food vacuoles than chloroquine-sensitive parasites. 3-Hydroxylpyridin-4-ones are known to chelate iron; hydroxypyridone-chloroquine (HPO-CQ) hybrids were synthesized in order to determine whether they can inhibit parasites proliferation in the parasitic digestive vacuole by withholding iron from plasmodial parasite metabolic pathway.

**Methods:**

Two HPO-CQ hybrids were tested against *Plasmodium falciparum* chloroquine-sensitive (D10 and 3D7) and -resistant strains (Dd2 and K1). The pharmacokinetic properties of active compounds were determined using a mouse model and blood samples were collected at different time intervals and analysed using LC–MS/MS. For in vivo efficacy the mice were infected with *Plasmodium berghei* in a 4-day Peters’ test. The parasitaemia was determined from day 3 and the course of the infection was followed by microscopic examination of stained blood films every 2–3 days until a rise in parasitaemia was observed in all test subjects.

**Results:**

IC_50_ values of the two compounds for sensitive and resistant strains were 0.064 and 0.047 µM (compound 1), 0.041 and 0.122 µM (compound 2) and 0.505 and 0.463 µM (compound 1), 0.089 and 0.076 µM (compound 2), respectively. Pharmacokinetic evaluation of these compounds showed low oral bioavailability and this affected in vivo efficacy when compounds were dosed orally. However, when dosed intravenously compound 1 showed a clearance rate of 28 ml/min/kg, an apparent volume of distribution of 20 l/kg and a half-life of 4.3 h. A reduction in parasitaemia was observed when compound 1 was dosed intravenously for four consecutive days in *P. berghei*-infected mice. However, a rise in parasitaemia levels was observed on day 6 and on day 9 for chloroquine-treated mice.

**Conclusion:**

The hybrid compounds that were tested were able to reduce parasitaemia levels in *P. berghei*-infected mice when dosed intravenously, but parasites recrudesced 24 h after the administration of the least dose. Despite low oral bioavailability, the IV data obtained suggests that further structural modifications may lead to the identification of more HPO-CQ hybrids with improved pharmacokinetic properties and in vivo efficacy.

## Background

Malaria is a disease caused by an eukaryote parasite of the genus *Plasmodium* and remains a major global public health problem, that was responsible for 584,000 deaths in 2013, with most occurring in Africa and most deaths in children under 5 years of age [1, 2]. *Plasmodium falciparum* and *Plasmodium vivax*, are responsible for most cases of malaria and the control of these parasites by chloroquine and other known anti-malarial drugs has been compromised by the emergence and spread of drug resistance in many parts of the world, primarily in *P. falciparum* strains [3, 4]. This has severely limited the use of many effective anti-malarials, and has become a major threat to malaria elimination efforts, causing increased morbidity and mortality and a financial burden due to sustenance of replacement therapy [3].

When the parasite infects the erythrocyte, it uses the endolysosomal system to digest the haemoglobin in an acidic food vacuole, producing an oxidized form of haem, ferriprotoporphyrin IX (FP-IX), which is the iron containing non-protein component of haemoglobin, as a by-product [5–7]. Free FP-IX is toxic and can lyse the cell and affect the function of lysozomal enzymes. The parasite disposes the toxic FP-IX by a polymerization process that crystallizes at least 95 % of FP-IX as haemozoin, and this allows an uninterrupted growth and proliferation of the parasite [8–10]. Iron chelators have been studied as alternative malaria drugs for many years because of their ability to interact with available iron in the nucleus and parasite cytosol, thereby interfering with the iron-dependent metabolism of malaria parasites and inhibiting their development [11, 12].

Hydroxypyridones are iron-chelating agents known to suppress malaria growth in vivo and in vitro [13, 14]. Both hydroxypyridones and chloroquine target the erythrocytic stage of the malaria life cycle, which is highly dependent on iron. For the purpose of this study, *N*-alkyl-3-hydroxypyridin-4-ones were combined with chloroquine in an attempt to enhance antiplasmodial effect against chloroquine-resistant strains of *P. falciparum* when compared to inhibition by chloroquine alone [13]. Compound 1 and 2 were selected among a series of hydroxypyridone-chloroquine (HPO-CQ) hybrid compounds in order to assess their antiplasmodial activity in vitro and efficacy in vivo and to evaluate their pharmacokinetic properties.

## Methods

### In vitro antiplasmodial activity against chloroquine-sensitive and resistant strains

The human parasite *P. falciparum* strains used in this study were chloroquine-sensitive (CQS) (3D7 and D10) and chloroquine-resistant (CQR) (Dd2 and K1) and were obtained from the malaria reagent depository, Malaria Research and Reference Reagent Resource Center (MR4) (ATCC, Manassas, VA, USA). The parasites were continuously cultured in vitro according to the method described by Trager and Jensen but with modifications [15]. The cultures were maintained at 37 °C in O-positive (O^+^) human erythrocytes in complete culture medium, which contained RPMI 1640 (Gibco-BRL Laboratories) growth medium supplemented with 25 mM 4-(2-hydroxyethyl)piperazine-1-ethanesulfonic acid (HEPES) (Sigma-Aldrich Chemical Company) buffer, 22 mM glucose (Sigma), 5 g/l albumax (Gibco-BRL Laboratories), 25 mM sodium carbonate (Sigma) and 0.3 mM hypoxanthine (Sigma). In order to control microbial contamination 50 µg/l gentamicin (Sigma) was added to the culture medium, and to obtain a loosely synchronous ring stage, 5 % sterile aqueous d-sorbitol (Sigma) was used. The antiplasmodial assay was initiated with the parasites in the trophozoite stage and with a parasitaemia and haematocrit of 2 %.

The test compounds were tested at a concentration range of 0.15–150 mM and chloroquine (Sigma) was tested at a concentration range of 0.003–3 and 0.0003–0.3 mM for resistant strain and sensitive strains, respectively. Non-parasitized erythrocytes were used as a negative control and parasitized erythrocytes, without any test compound, were used as positive control. A full day dose–response was performed for all compounds to determine the concentration inhibiting 50 % of parasite growth (IC_50_ value). The plates were incubated for 48 h at 37 °C in a gassing chamber containing a mixture of 5 % CO_2_, 3 % O_2_ and 92 % N_3_. In order to quantify parasite viability and to determine the effect of the compounds on the parasite, a parasite lactate dehydrogenase (pLDH) assay was used as described by Makler et al. [16, 17], but with modifications.

### Pharmacokinetic studies

A comprehensive pharmacokinetic (PK) study of compound 1 and 2 was performed on 10-week old male and female C57BL/6 mice (20–30 g) obtained from the University of Cape Town Medical School Animal Unit. The mice were housed in ventilated cages at room temperature (approximately 22 °C) with constant supply of food and water and were monitored twice daily. The study was authorized by the Faculty of Health Science Animal Research Ethics Committee before commencement: Reference No. 012/020. All the work was performed according to the guidelines established by Austin et al. [18].

The compounds were prepared in oil-in-water (o/w) microemulsions, which consist of 5 % ethyl linoleate (Sigma), 11 % Tween 80 (Sigma), 4 % ethanol (Merck), and 80 % water. In each experiment a group of five animals were dosed orally (10 ml/kg) at 20 mg/kg and intravenously (5 ml/kg) via the dorsal penile vein at 4 mg/kg under anaesthesia. For oral dosage, a gavage needle was used for the administration of test compounds directly into the lower oesophagus or stomach. Blood samples (approximately 30 µl) were collected serially by needle prick on the tail vein, near to the tip of the tail, at 0, 0.17, 0.5, 1, 2, 3, 5, 7, and 9 h post-dosing. Lithium heparin-coated MiniCollect^®^ Plasma Tubes (Lasec, South Africa) were used to collect the blood samples. The collected blood samples were placed on ice immediately after sampling, and were frozen at −80 °C until analysis.

### Sample preparation

The blood samples stored at −80 °C were thawed at room temperature and then mixed by vortex to ensure homogeneity. Twenty µl of blood was mixed with 50 µl Milli-Q water (Millipore, USA) and 150 µl acetonitrile (Merck). The mixture was vortexed for 15 s, sonicated for 10 min and centrifuged at 13,000*g* for 5 min. The supernatant was transferred to a flat-bottomed glass insert and placed in a glass vial and placed in the autosampler for analysis.

### Liquid chromatography–mass spectrometry summary

A liquid chromatography–mass spectrometry **(**LC/MS/MS) system was employed for the quantification of the compounds in mouse blood. The LC system employed was an ultra-fast liquid chromatography (UFLC) system (Shimadzu) and the separation of the compounds was performed on a Phenomenex, Luna 5 μm PFP (2), 100 Å, 50 mm × 2 mm analytical column. The mobile phase A consisted of 0.1 % formic acid in water (v/v) and mobile phase B consisted of acetonitrile. The flow rate was set at 500 µl/min and the temperature of the column was maintained at 40 °C. For the separation of the compounds, the mobile phase was increased from 5 to 95 % B over 4 min, after that, phase B was returned to 5 % within 0.1 min, then equilibrated for 3 min.

The detection of the compounds was performed on an AB Sciex 3200 Q-Trap mass spectrometer which was operated at unit resolution in the multiple reaction monitoring (MRM) mode. The calibration range for all the compounds was between 7.8 and 1000 ng/ml and the accuracy (% Nom) for the calibration curves was between 90.3 and 104.0 %. Table 1 gives an overview of the MS parameters and the instrument settings.

**Table 1:** Mass spectrometer settings and MS parameters used for the detection of the test compounds on an API 3200 Q-Trap

Parameter	Compound 1	Compound 2
Q1 mass (Da)	531.2	490.2
Q3 mass (Da)	91.1	261.2
Dwell time (ms)	40	40
Declustering potential (V)	91	61
Collision energy (V)	89	73
Entrance potential (V)	10	10
Collision cell exit potential (V)	4	2
Source temperature (°C)	500	500
Curtain gas (psi)	25	25
Gas 1 (psi)	50	50
Gas 2 (psi)	70	70
CAD gas	Medium	Medium
Ion spray voltage (kV)	5500	5500
Ionization mode	Positive	Positive

### Pharmacokinetic analysis

Non-compartmental analysis was performed on each individual set of data using PK Solutions 2.0 Pharmacokinetic Analysis Software (Summit Research Services, Montrose, USA). The following PK parameters were calculated: apparent terminal half-life [t½ (min)], total exposure [AUC_0–α_ (µM min)], volume of distribution (l/kg) and plasma clearance [CL (l/min/kg)].

### In vivo anti-malarial efficacy of compound 1 against a chloroquine-sensitive *Plasmodium berghei* strain

The chloroquine-sensitive *Plasmodium berghei* (ANKA strain) was used to assess in vivo anti-malarial efficacy of the test compounds. The parasites were maintained in a C57BL/6 mouse by inoculation with 250 µl of a 1:1 (v/v) suspension of erythrocytes infected with *P. berghei* in phosphate buffered saline (PBS). On the day of the experiment the host mouse was anaesthetized intraperitoneally with a mixture of ketamine (120 mg/kg) and xylazine (16 mg/kg). Whole blood from the host mouse was drawn by cardiac puncture into a Vacuette^®^ heparin tube and a suspension of *P. berghei* parasitized erythrocytes (1 × 10^7^) in PBS was prepared and the test mice were infected with 200 µl of this suspension intraperitoneally.

Evaluation of the curative potential of the test compounds was performed using Peters’ 4-day test as described [19]. The mice were dosed orally at 20 and 40 mg/kg and intravenously at 4 and 8 mg/kg 2 h post-infection and for three consecutive days (D_0_ to D_3_). On the first day (D_0_), blood samples were collected serially from each mouse at 0.5, 1, 3, and 7 h post-dosing in order to provide quantitative measurements of drug exposure which is needed for the sound interpretation of the efficacy of the anti-malarials [20]. Chloroquine was used as a reference drug and was dosed orally at 10 mg/kg. The mice were also dosed orally with PBS as a control. The parasitaemia was determined from the third day (D_4_) by preparing thin blood films from the tail of each mouse and the smears were fixed with methanol and stained with Giemsa. This was repeated every 2–3 days to monitor the efficacy of the test compounds. The formula used to calculate % parasitaemia and % reduction is described below, respectively. The data for % parasitaemia and % reduction of parasitaemia is presented in Table 4.

$$ \% \,\,{\text{Parasitaemia = }}\frac{{{\text{No}} .\,\,{\text{of}}\,\,{\text{parasitized}}\,\,{\text{RBC}}\,\,{\text{out}}\,\,{\text{of}}\,\, 5 0 0\,\,{\text{erythrocytes}}}}{{{\text{Total}}\,\,{\text{no}} .\,\,{\text{of}}\,\,{\text{RBC}}\,\,{\text{counted}}}} \times 100 $$

$$ \% \,\,{\text{Reduction = }}\frac{{{\text{Parasitamia}}\,\,{\text{of}}\,\,{\text{placebo }}- {\rm Parasitamia}\,\,{\text{of}}\,\,{\text{test}}\,\,{\text{compound}}}}{{{\text{Parasitamia}}\,\,{\text{of}}\,\,{\text{placebo}}}} \times 100 $$

## Results

### Antiplasmodial action of the compounds in vitro

Compounds 1 and 2 were synthesized in order to enhance the activity of chloroquine by combining it with an iron chelator. These compounds were tested for antiplasmodial activity against sensitive strains 3D7 and D10 and resistant strains K1 and Dd2 and the IC_50_ values (in µM) of compounds 1 and 2 are summarized in Table 2. The *P* values were calculated using the non-parametric Mann–Whitney U test in order to compare the activity of chloroquine with that of the HPO-CQ hybrids. The resistance index (RI), the ratio of the IC_50_ of the resistant strain to that of the sensitive strain, was calculated for these compounds using the lowest IC_50_ value of the sensitive and the highest IC_50_ of the resistant strain for each compound, which should give the highest RI value for that compound [21]. This value provides a quantitative measurement of the antiplasmodial activity against CQR strains relative to that against CQS strains [22]. The higher the RI value, the higher the level of resistance. The RI value was calculated according to this formula [23]:

$$ {\text{Resistance}}\,\,{\text{Index}}\,\, ( {\text{RI) = }}\frac{{{\text{Chloroquine}} - {\text{resistant}}\,\,{\text{strain}}\,\,{\text{IC}}_{50} }}{{{\text{Chloroquine}} - {\text{sensitive}}\,\,{\text{strain}}\,\,{\text{IC}}_{50} }} $$

**Table 2:** In vitro IC_50_ values (µM) of compounds 1 and 2

Test compounds	Sensitive strains		Resistant strains		RI	*P* value
	3D7	D10	K1	Dd2		
1	0.064 ± 0.02	0.047 ± 0.01	0.505 ± 0.10	0.463 ± 0.12	10.7	0.343
2	0.041 ± 0.02	0.122 ± 0.03	0.089 ± 0.01	0.076 ± 0.01	2.2	1
Chloroquine	0.019 ± 0.00	0.023 ± 0.00	0.279 ± 0.002	0.180 ± 0.01	14.4	n/a

The values obtained from antiplasmodial tests represent the mean of three independent experiments each performed in triplicate

RI value, values calculated using the highest CQR IC_50_ value and lowest CQS IC_50_ value; n/a, not applicable

**Fig. 1:**
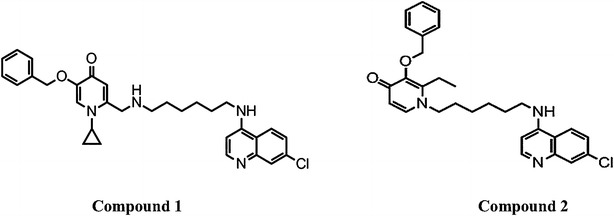
The structures of synthesized hydroxypyridone-chloroquine hybrids (compound 1 and 2)

The IC_50_ values calculated from the dose response curves were in the range of 0.041–0.064 µM against 3D7 and 0.047–0.122 µM against D10. These values were higher than that of chloroquine, with IC_50_ values of 0.019 and 0.023 µM against 3D7 and D10, respectively. The IC_50_ values of the compounds against the resistant strain K1 and Dd2 were compared to those of chloroquine (IC_50_ values of 0.279 and 0.180 µM for K1 and Dd2, respectively). Compound 2 was more active than chloroquine with IC_50_ values of 0.089 and 0.076 µM for K1 and Dd2, respectively. Compound 1 on the other hand was less active than chloroquine with IC_50_ values of 0.505 and 0.463 µM for K1 and Dd2, respectively. The data presented in Table 2 show that the difference in the activity of the HPO-CQ hybrids when compared to chloroquine is insignificant at *P* > 0.05 in all chloroquine-sensitive strains. However, activity of the two compounds against the resistant strains differed significantly. The compounds had lower RI values than chloroquine. Compound 2 had an RI value of 2.2, which is over five times less than that of chloroquine and the RI value of compound 1 was 10.7 (Fig. 1).

### Pharmacokinetics

**Fig. 2:**
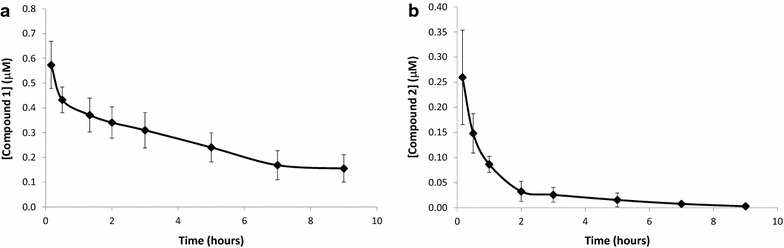
Blood concentrations of compounds 1 (**a**) and 2 (**b**) in C57BL/6 mice blood after intravenous administration of 4 mg/kg. Data represent mean ± standard deviation of data points obtained from five single mice

The compounds were administered to mice orally and intravenously at 20 and 4 mg/kg, respectively. The blood levels for both compounds in the mice dosed orally could not be detected because the values were below the limit of quantitation (LOQ). The concentration versus time intravenously (IV) profiles of both compounds is presented in Fig. 2. The mean concentration–time data were used to calculate the PK parameters by non-compartmental analysis using PK Solutions 2.0 Pharmacokinetic Analysis Software. The PK parameters are presented in Table 3.

**Table 3:** Pharmacokinetic parameters of hydroxypyridone-chloroquine hybrids after intravenous administration

Parameters	Compound 1	Compound 2
T_1/2_ (h)	4.3 ± 1.02	0.7 ± 0.2
Vss (l/kg)	20.1 ± 6	154.1 ± 1
CL (ml/min/kg)	27.6 ± 8	366 ± 46.6
AUC_0–∞_ (µM min)	196 ± 29.9	25.2 ± 3.4

Data represent mean ± standard deviation of data points obtained from five uninfected mice

*ND* indicate that the value was not determined

Compound **1** showed a clearance rate of 28 ml/min/kg, a high apparent volume of distribution of 20 l/kg and a half-life of 4.3 h. The area under the curve (AUC) value was also at 196 µM min. Even with the half-life of 4.3 h, the compound remained at detectable levels of 0.16 µM after 9 h at the end of the dosing interval, indicating a slow elimination rate from circulation. When considering these properties and IC_50_ values of 0.064–0.505 µM against sensitive and resistant strains, respectively, compound 1 was selected for in vivo efficacy studies. Even though the compound could not be detected in blood following an oral dosage, investigating the efficacy of compound 1 after an intravenous dosage was considered.

Compound 2, on the other hand, had a very short half life of 0.7 h and after 2 h the blood levels dropped to 0.03 µM because of a high clearance rate of 366 ml/min/kg. The volume of distribution was also high at 154.1 l/kg.

### Anti-malarial effect of compound 1 on *Plasmodium berghei*-infected mice

**Fig. 3:**
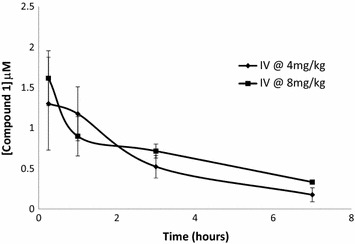
Blood levels of compound 1 in C57BL/6 mice blood infected with *P. berghei* after intravenous administration of 4 and 8 mg/kg of compound. Data represent mean ± standard deviation of data points obtained from five mice

The PK evaluation shows that compound 1 and 2 levels were below the LOQ. They both presented poor PK properties following oral administration. However, the LC–MS/MS assay only detected the parent compound and no assays were conducted to measure or identify any possible metabolites. The decision to perform efficacy studies was based on the possibility that compounds 1 and 2 may have high first pass effect which may cause low bioavailability. After the infected mice were dosed orally and intravenously with compound 1 and 2, the blood concentrations were measured in *P. berghei*-infected mice at different time intervals to measure the exposure of the infected mice to the drug. The blood concentration levels of the mice that were dosed orally were below the detection level in both 20 and 40 mg/kg groups. Figure 3 shows concentration versus time profiles of compound 1 following IV administration in mice infected with *P. berghei*. For compound 2 the IV data showed a very high clearance rate compared to compound 1 and blood concentration levels were very low. High toxicity was also detected when dosed orally and intravenously. The PK studies were only conducted for 9 h, and therefore the symptoms of toxicity were not severe. However, with efficacy studies the mice are dosed every day for 4 days, and this might cause distress to the mice because the toxic effect of the drug will be more severe. The efficacy study for compound 2 is not reported in this paper.

When considering compound 1 blood concentration of healthy animals in Fig. 2a and *P. berghei*-infected animals in Fig. 3, it was noted that higher drug levels were observed in infected mice. It is unlikely that the presence of parasites could be the reason for such a significant difference in blood levels because the mouse exposure study was performed 2 h post infection and parasitized erythrocytes are rapidly absorbed after intraperitoneal inoculation [24]. This difference could possibly be due to the variation in age and weight.

To evaluate the anti-malarial efficacy of compound 1 in infected mice, the parasitaemia level was calculated at days 4, 6 and 9 post infection, and the % reduction of parasitaemia was calculated for day 9 only because parasite levels were observed in all treated mice on day 9.

In the mice that were dosed orally, the parasites were visible at a parasitaemia of ±5 % on day 4. These data correlate well with the PK data because the blood levels of compound 1 following oral dosage were too low to have any effect on reducing parasitaemia, and thus allowing the disease to progress as indicated by a rise in the parasitaemia levels, as shown in Table 4. It was also noted that the parasitaemia in mice that were dosed orally was higher than the parasitaemia in the placebo group. This may mean that high oral doses may cause compound 1 to be immunosuppressive, thus allowing the infection to be severe in the oral group when compared to the placebo group. High parasitemia levels in the 40 mg/kg/day oral group resulted in a negative value of % reduction of parasitaemia (Table 4).

**Table 4:** In vivo antiplasmodial efficacy of compounds 1 in C57BL/6 mice blood infected with *P. berghei*

Test group	Average % parasitemia			% reduction of parasitemia
	Day 4	Day 6	Day 9	Day 9
Oral (20 mg/kg/day)	5.3 ± 3.9	22.7 ± 10	24.2 ± 5.6	3.7 ± 5.6
Oral (40 mg/kg/day)	5.9 ± 2	10.7 ± 5	48.6 ± 12	−93.8 ± 49.3
IV (4 mg/kg/day)	0.4 ± 0.4	1.8 ± 1.4	8.1 ± 5	61.9 ± 26
IV (8 mg/kg/day)	0	0.9 ± 0.9	5.1 ± 1.7	82.9 ± 2.5
Oral CQ (10 mg/kg/day)	0	0	2 ± 1.6	92.0 ± 0.4
Placebo	2.5 ± 2.4	7.3 ± 2.4	25.1 ± 0.4	–

Data represent mean ± standard data points obtained from five mice

As expected, blood levels remained high for a longer period for mice dosed intravenously with 4 and 8 mg/kg of compound 1 as shown in the concentration versus time profiles in Fig. 3. These data are consistent with the comprehensive PK study presented in Table 3, which shows that compound 1 had a half-life of 4.3 h following intravenous dosage and a clearance rate of 28 ml/min/kg. Figure 3 shows that after 7 h following intravenous dosage the blood concentration levels were relatively high for 4 and 8 mg/kg. This resulted in a reduced parasite multiplication rate as indicated by undetected to low parasitaemia until day 4 and 6. However, the data suggest that compound 1 suppressed the parasitaemia without clearing all parasites, therefore, surviving parasites in the mice blood began to multiply after the 4-day course of treatment, causing a parasite reduction to decrease to 62 and 83 % in the mice dosed at 4 and 8 mg/kg on day 9, respectively, as shown in Table 4. This also shows that the efficacy of compound 1 is dose dependent.

## Conclusion

The aim of synthesizing HPO-CQ hybrids was to develop anti-malarial candidates that were potent against sensitive and resistant *P. falciparum* strains. This study showed that combining chloroquine with a HPO can cause a change in the physicochemical properties of the hybrid, thus affecting its efficacy and PK properties. Chloroquine alone has an oral bioavailability of 79–90 % and a half-life of 7 h in mice, as reported by Salako [25]. Even though compound 2 was more active than chloroquine in vitro against the resistant strains with a lower RI value, the PK properties were not improved relative to chloroquine. Compound 1 was less active in vitro against the resistant strains than compound 2 and chloroquine with poor oral bioavailability, but it proved to be an effective anti-malarial compound in vivo when dosed intravenously. Nevertheless, chloroquine remained more effective in vivo than both compounds and this means that further modifications on the chemical structure could improve PK properties, such as oral bioavailability, thus improving efficacy.

## Abbreviations

AUC: area under the curve
CL: clearance
CQ: chloroquine
CQR: chloroquine resistant
CQS: chloroquine sensitive
Da: Daltons
FP-IX: ferriprotoporphyrin IX
HEPES: 4-(2-hydroxyethyl)piperazine-1-ethanesulfonic acid
HPO: hydroxypyridones
IV: intravenously
LC–MS/MS: liquid chromatography–mass spectrometry
LOQ: limit of quantitation
MRM: multiple reaction monitoring
PBS: phosphate buffered saline
PK: pharmacokinetics
pLDH: lactate dehydrogenase
RBC: red blood cells
RI: resistance index
T_1/2_: half-life
UFLC: ultra-fast liquid chromatography
Vss: volume of distribution
